# Surface-Electromyography-Based Co-Contraction Index for Monitoring Upper Limb Improvements in Post-Stroke Rehabilitation: A Pilot Randomized Controlled Trial Secondary Analysis

**DOI:** 10.3390/s23177320

**Published:** 2023-08-22

**Authors:** Virginia Bandini, Ilaria Carpinella, Alberto Marzegan, Johanna Jonsdottir, Carlo Albino Frigo, Laura Avanzino, Elisa Pelosin, Maurizio Ferrarin, Tiziana Lencioni

**Affiliations:** 1 IRCCS Fondazione Don Carlo Gnocchi Onlus, Via Capecelatro 66, 20148 Milan, Italy; vbandini@dongnocchi.it (V.B.); icarpinella@dongnocchi.it (I.C.); amarzegan@dongnocchi.it (A.M.); jjonsdottir@dongnocchi.it (J.J.); tlencioni@dongnocchi.it (T.L.); 2 Department of Electronics, Information and Bioengineering (DEIB), Politecnico di Milano, Piazza Leonardo da Vinci 32, 20133 Milan, Italy; carlo.frigo@polimi.it; 3 Department of Experimental Medicine, Section of Human Physiology, University of Genoa, 16132 Genoa, Italy; laura.avanzino@unige.it; 4 IRCCS Ospedale Policlinico San Martino, IRCCS, 16132 Genoa, Italy; elisa.pelosin@unige.it; 5 Department of Neuroscience, Rehabilitation, Ophthalmology, Genetics and Maternal Child Health, University of Genoa, 16132 Genova, Italy

**Keywords:** stroke, sEMG analysis, muscle co-contraction, *CCI*, rehabilitation, upper limb assessment

## Abstract

Persons post-stroke experience excessive muscle co-contraction, and consequently the arm functions are compromised during the activities of daily living. Therefore, identifying instrumental outcome measures able to detect the motor strategy adopted after a stroke is a primary clinical goal. Accordingly, this study aims at verifying whether the surface electromyography (sEMG)-based co-contraction index (*CCI*) could be a new clinically feasible approach for assessing and monitoring patients’ motor performance. Thirty-four persons post-stroke underwent clinical assessment and upper extremity kinematic analysis, including sEMG recordings. The participants were randomized into two treatment groups (robot and usual care groups). Ten healthy subjects provided a normative reference (NR). Frost’s *CCI* was used to quantify the muscle co-contraction of three different agonist/antagonist muscle pairs during an object-placing task. Persons post-stroke showed excessive muscle co-contraction (mean (95% CI): anterior/posterior deltoid *CCI*: 0.38 (0.34–0.41) *p* = 0.03; triceps/biceps *CCI*: 0.46 (0.41–0.50) *p* = 0.01) compared to NR (anterior/posterior deltoid *CCI*: 0.29 (0.21–0.36); triceps/biceps *CCI*: 0.34 (0.30–0.39)). After robot therapy, persons post-stroke exhibited a greater improvement (i.e., reduced *CCI*) in proximal motor control (anterior/posterior deltoid change score of *CCI*: −0.02 (−0.07–0.02) *p* = 0.05) compared to usual care therapy (0.04 (0.00–0.09)). Finally, the findings of the present study indicate that the sEMG-based *CCI* could be a valuable tool in clinical practice.

## 1. Introduction

Stroke is one of the major causes of long-term disability worldwide, and stroke survivors typically have difficulty performing activities of daily living (ADLs) [1]. The impairment of the paretic arm is one of the most disabling post-stroke motor consequences [2], which includes an exaggerated muscle co-contraction (simultaneous activity of agonist and antagonist muscles) [3,4,5,6,7,8].

Agonist-antagonist muscle co-contraction is an essential and common motor control strategy used by the central nervous system to regulate joint stability and provide movement accuracy [9,10]. After a stroke, there is an alteration in these coordination patterns, resulting in motor deficits of the arm function [4]. Specifically, excessive muscle co-contraction after neurological injuries can lead to impaired movement due to muscle weakness [10], and increased physiological, metabolic, and energetic costs [9,11,12,13]. These alterations consequently cause a reduction in the net moment and power development [11], which are functionally disadvantageous and result in the inefficacy of human movement [11]. Therefore, the reduction in the exaggerated muscle co-contraction and subsequent recovery of more physiological patterns of muscle activation might lead to an improvement in upper limb functionality of persons post-stroke [14].

The identification of instrumented outcome measures, able to characterize the neural strategy adopted after a stroke, can be useful in clinical practice for a better understanding of how the disease modifies muscle strategies during the performance of movements, allowing the planning of personalized training [12] and monitoring the effect of treatment.

Surface electromyography (sEMG) records the electrical activity of muscles via electrodes placed over the skin, and it is a suitable tool for detecting antagonist muscle co-contraction, allowing to distinguish between physiological and pathological patterns [15,16,17,18,19,20,21].

In fact, for more than five decades, sEMG has been employed in the neurorehabilitation field as a tool to assess muscle activation and improve neuromuscular rehabilitation treatments [17,18]. For example, the EMG-based analysis has been used to investigate interlimb coordination, provide biofeedback, and track rehabilitation responses in subjects with neurological impairments [22]. In addition, the potential of sEMG in prognosticating recovery is clinically recognized, as it provides specific quantitative evidence for treatment decisions [22].

However, although the literature has already provided substantial evidence that sEMG applications supply relevant information in the neurorehabilitation area to enhance motor recovery, sEMG-based outcome measures currently have limited use in clinical practice [15,22].

Appropriate interpretation of sEMG needs both technical and clinical expertise since sEMG parameters extracted from the signal are typically obtained by means of complex methods [23]. Therefore, the availability of simplified and intuitive EMG-based analysis, provided that it correctly represents muscle recruitment, could facilitate the clinical application of sEMG in clinical settings [22].

The sEMG-based co-contraction index (*CCI*), which is commonly used for the quantification of muscle co-contraction [4,8,9], can be an easy-to-use method for assessing motor control, having a quick implementation and easy interpretation even by non-technical experts, such as clinicians [9]. Previous studies have proposed Frost’s *CCI* as a quantitative, robust, and accurate outcome measure in pathological subjects, capable of providing a rapid indication of the simultaneous activation of antagonist muscles with respect to healthy subjects [4,13,24,25,26]. The use of such an instrumented index overcomes the limitations related to clinical scales as outcome measures, which may suffer from poor sensitivity to changes and floor/ceiling effects.

We have already investigated the changes induced by rehabilitation on the patterns of muscle synergies activation using complex techniques of clustering, which decompose multiple sEMG signals into a limited number of primitive patterns [2]. However, the interpretation of the muscle synergies parameters is quite difficult and requires a close collaboration between clinicians and engineers, which makes it difficult to transfer such a procedure to clinical practice [15,22]. Consequently, the development of easy-to-use methods for assessing motor control in a clinical setting could be valuable in tailoring the treatment of persons post-stroke. This is a topic of particular interest in the clinical field, but a recent review highlighted that sEMG is a promising tool to further improve functional recovery and stressed the need to develop specific sEMG-based interventions [17].

This study aims at investigating whether an sEMG-based *CCI*, namely Frost’s *CCI*, which is a direct expression of muscular activity, can be an outcome measure able to detect (1) the deviation of the pathological muscle activation pattern from the physiological one, and (2) the rehabilitation-induced changes in the upper limb of persons post-stroke, setting a new clinically feasible approach in the evaluation of patients’ motor performance.

## 2. Materials and Methods

### 2.1. Participants

Persons post-stroke were recruited from the Neurorehabilitation Department of IRCCS Don C. Gnocchi Foundation (Milan, Italy) between March 2015 and November 2017. The inclusion criteria were as follows [1,2]: (i) age > 18 years, (ii) first-time ischemic or hemorrhagic stroke, (iii) a National Institute of Health Stroke Scale Motor Arm score ranging from 1 to 3, and (iv) a score higher than 6 out of 66 points on the Fugl-Meyer Motor Assessment of Upper Extremity (FM-UE) scale. The exclusion criteria were as follows [1,2]: (i) the presence of a moderate cognitive decline with a Mini-Mental State Examination score of <20 points, (ii) evidence of severe verbal comprehension deficit, apraxia, and/or visuospatial neglect (iii) evidence of behavioral disturbances (i.e., delusions, aggressiveness, and severe apathy/depression), which could affect compliance with the rehabilitation programs, (iv) presence of non-stabilized fractures, (v) presence of traumatic brain injury and (vi) presence of drug-resistant epilepsy.

A sample of 10 healthy subjects without any neurological or musculoskeletal disorders provided the normative reference.

### 2.2. Study Design

This study was a secondary analysis of the MOSE (Modularity for Sensory Motor Control) study (ClinicalTrial.gov, NCT03530358), a larger bi-center randomized controlled trial, and analyzed the data recorded at the IRCCS Don C. Gnocchi Foundation, Milan, Italy (Center 2), which tested the robot-assisted therapy for the upper limb rehabilitation after stroke [1,2].

The study design, in both centers, consisted of a single-blind two-arm randomized 1:1 controlled trial. The subjects’ randomization was performed using a computerized automated program and was stratified according to disease onset (≤3 months or >3 months) to ensure group comparability in terms of the number and chronicity of participants.

### 2.3. Rehabilitation Program

The rehabilitation program for the paretic arm for both treatment groups (robot group–RG and usual care group–UCG) consisted of 20 45 min sessions, carried out 5 times a week by trained physiotherapists. The interventions have been already described in previously published papers [1,2] and are briefly reported below.

The RG treatment consisted of a planar robotic manipulandum (Braccio Di Ferro, Celin s.r.l., Italy), as fully described in the literature [27]. The robotic system was designed to practice shoulder and elbow mobility of the paretic arm in the horizontal plane by controlling the position of the robot end-effector. During the training, subjects were asked to perform repeated center-out reaching movements and back from a central position to five peripheral spots randomly placed on a semicircle with a radius of 20 cm. The robot device could work in two different force modalities, assist-as-needed or resistive, which were chosen by the physiotherapist in each session depending on the subject’s abilities.

Participants allocated to the UCG underwent conventional arm-specific physiotherapy consisting of sessions of active and passive mobilization of the shoulder, scapula, elbow, and wrist, followed by task-oriented exercises customized to the skills of the participants. Treatment progression was achieved by increasing the range of motion, the number of repetitions, and muscular coordination demands.

### 2.4. Outcome Measures

#### 2.4.1. Clinical Assessment

All participants post-stroke were clinically assessed by a qualified examiner, who was unaware of group allocation, at baseline (*T*0) and post-training (*T*1) using the FM-UE. FM-UE assessed the motor function of the paretic arm on a 3-point ordinal scale (0 = cannot perform; 1 = performs partially; and 2 = performs fully), applied to each of the scale items. Item scores were then summed to provide an overall score ranging from 0 (hemiplegia/severe impairment) to 66 (normal motor performance/no impairment).

#### 2.4.2. Instrumented Assessment

**Figure 1:**
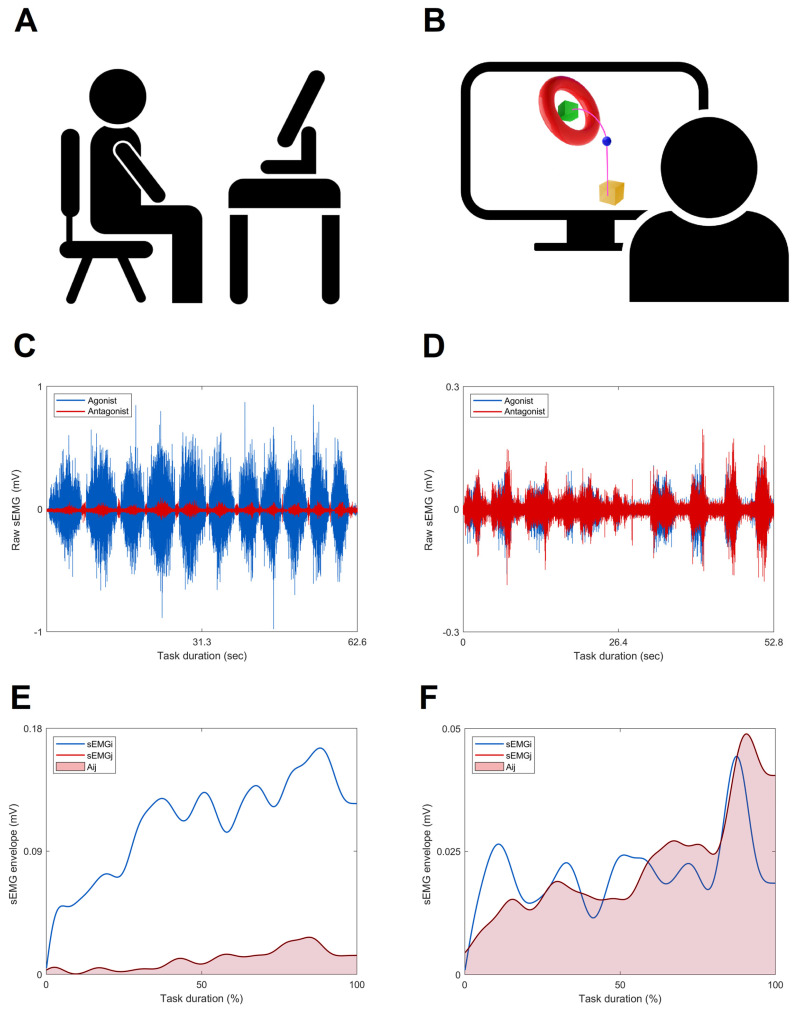
The upper panel shows a schematic representation of the experimental set-up during the performance of a subject (**A**,**B**). The blue ball indicates the hand’s movement, the yellow box the rest position, and the green cube the final position. The purple line shows the hypothetical trajectory of a representative subject (not shown during the test). The middle panel reports examples of the raw sEMG signals of the agonist and antagonist muscles (anterior deltoid, blue line, and posterior deltoid, red line) during the object-placing task of a healthy subject (**C**) and a person post-stroke (**D**). The lower panel shows the normalized envelope of the muscle pairs’ anterior and posterior deltoids in a single repetition for a healthy subject (**E**) and a person post-stroke (**F**). The respective overlapping area is highlighted in light red (Aij, red area).

All persons post-stroke were asked to perform a 3D functional motor task, named the object placing task, with the paretic arm. This task simulated a typical activity of daily living and was recorded at both *T*0 and *T*1 to evaluate the effects of rehabilitation treatments during an untrained functional task. In the resting position, the participants sat on a chair in front of a screen with both feet resting on the floor, the knees and hips bent at 90 degrees, and both hands placed at mid-thigh. During the execution of the task, subjects held an electromagnetic sensor with the paretic hand and received visual feedback of the performance in a virtual reality scenario (VRRS—Virtual Reality Rehabilitation System, Khymeia Group Ltd., Padova, Italy) (Figure 1A). Virtual objects were displayed on the screen representing the resting position (i.e., yellow box), the hand’s movements (i.e., blue ball), and the final position (i.e., green box), and the subjects were asked to move the virtual ball from the initial to the final position at their preferred velocity (Figure 1B).

The experimental setup and equipment have been previously described in detail [1,2]. Briefly, a 9-camera optoelectronic system (SMART-DX, BTS Bioengineering, Garbagnate Milanese, Italy) acquired the trajectory of the hand’s marker (10 mm diameter) of the tested limb with a sample rate of 200 Hz and low-pass filtered at 6 Hz.

Muscle activity was recorded using a wireless multichannel sEMG system (CometaWavePlus, Cometa s.r.l., Bareggio, Italy). Surface electrodes were placed according to the SENIAM (Surface Electromyography for the Non-Invasive Assessment of Muscles) specifications [28] and anatomical guidelines [29]. The activities of the following 6 muscles of the paretic limb, corresponding to three pairs of antagonistic muscles were recorded: (1) anterior deltoid (ANDE, agonist) and posterior deltoid (PODE, antagonist); (2) triceps brachii lateral head (TBLH, agonist) and biceps brachii short head (BBSH, antagonist); and (3) pronator teres (PRON, agonist) and supinator teres (SUPI, antagonist). The sEMG frequency of acquisition was 1000 Hz.

The instants of initiation and termination of each repetition of the object pacing task were computed as the times at which the velocity of the hand marker exceeded or fell below a threshold of 5% of the maximum value [1,2]. Raw sEMG signals (examples reported in Figure 1C,D) were band-pass filtered (10–400 Hz, 2nd order Butterworth), full-wave rectified and then low-pass filtered (4 Hz, 4th order Butterworth) in order to obtain the sEMG linear envelopes [24]. The sEMG linear envelopes were time-normalized to 100% of the movement duration, and then, to preserve the variability in sEMG, the signal of each muscle was amplitude-normalized to their maximum peak value obtained on all the recorded tasks [30].

#### 2.4.3. *CCI* Computation

Frost’s *CCI* (Equation (1)) was computed to quantify the muscle co-contraction of the three antagonistic muscle pairs recorded using sEMG. This approach required an a priori classification of muscle antagonists or agonists, depending on the generated movement [31]. During physiological movements, the agonist muscle exerted force and/or moment of force in the main direction defined by the task, whereas the antagonist muscle opposed this action [8,31].

(1) $$CCI_{Frost}=\frac{1}{T}\int_{0}^{T}A_{ij}\left(t\right)dt$$

In this expression (Equation (1)), proposed by Frost in 1997, $A_{ij}\left(t\right)$ represents the overlapping activity of the muscles *i* and *j* in the sEMG envelopes, while *T* represents the task duration of the signal (Figure 1E,F) [4,13,24,25,26]. The *CCI* ranges from 0 to 1 (dimensionless value (um)), where 0 indicates that the activities of the two muscles did not overlap at all during the task, while 1 indicates that the activities of the two muscles were fully overlapping, and the level of sEMG activity was maintained at 1 during the task [4,25]. Therefore, a higher value of the *CCI* indicates a greater muscle co-contraction and is classified using terms such as normal, increased, or reduced compared to the normative reference [32].

The *CCI* was computed for each trial and averaged across the repetitions for each participant at both *T*0 and *T*1.

#### 2.4.4. Kinematic Variables Quantification

Movement kinematics were assessed by calculating the speed and smoothness parameters for each trial and averaging them across the repetitions for each participant at both *T*0 and *T*1.

Movement speed was calculated as the distance traveled by the hand marker during a movement trial divided by the time employed for that movement.

Movement smoothness was calculated as the number of movement units (number of peaks) of the hand marker tangential velocity profile divided by the hand marker trajectory distance for each movement trial [33,34,35].

### 2.5. Statistics

#### 2.5.1. Sample Size Estimation

As reported in our previous studies [1,2], the sample size was estimated using the shoulder/elbow coordination kinematic index (CKI) as the primary outcome measure. Based on the change scores of the CKI with a Cohen’s d effect size of 1.40 between robot and control training previously published in the literature [36], a sample size of 24 subjects (12 per group) was calculated to detect a difference between the groups with α = 0.05 and power (1 − β) = 0.9. The previous overestimation (i.e., 20 per group) allowed us to obtain an adequate sample size for the analysis presented here. In fact, the guidelines for demonstration-of-concept pilot RCT studies on motor rehabilitation, such as the present study, report 15 subjects per group as the minimum sample size [37].

#### 2.5.2. Statistical Analyses

Statistical analyses were performed using SPSS and JASP. In the presence of normally distributed data, parametric tests were performed and data were reported as mean and 95% confidence interval (CI), whereas in case of deviation from normality, non-parametric tests were chosen and data were presented as median and interquartile range or percentage.

The demographic and clinical baseline scores of persons post-stroke were compared between the two groups of treatment (RG vs. UCG). Chi-square tests were utilized to compare sex, stroke type, paretic side, and chronicity. Time since stroke, age, and FM-UE scores at *T*0 (pre-treatment) were analyzed using the Mann–Whitney U test (RG vs. UCG).

The differences in muscle co-contraction and movement kinematics of the upper limb between the healthy subjects (NR) and persons post-stroke were analyzed using an unpaired *t*-test of *CCI* and speed and smoothness at *T*0.

To assess the rehabilitation-induced changes (RG vs. UCG) in the muscle co-contraction and in movement kinematics of the paretic arm of persons post-stroke, the change scores (CS, post–pre) of *CCI* and speed and smoothness were compared by ANCOVA using values at *T*0 as covariates. Since persons post-stroke showed higher *CCI* values with respect to the normative reference at *T*0, values of the *CCI* post-training lower than pre-training (CS < 0) were interpreted as an improvement in upper limb functionality (i.e., reduction in muscles co-contraction), while higher post-training values (CS > 0) were interpreted as a worsening.

A non-parametric ANCOVA analysis (Quade’s test) of the FM-UE CS was also applied to evaluate clinical changes following rehabilitation using the FM-UE score at *T*0 as a covariate.

Spearman’s correlation analysis was applied to analyze the correlation between the change in scores of the FM-UE clinical scale and the *CCI*.

Between-group differences (PA vs. NR and RG vs. UCG) and effect sizes (Cohen’s d) were also computed. Cohen’s d values of 0.2, 0.5, and 0.8 represented small, moderate, and large effect sizes, respectively [38].

*p*-values less than or equal to 0.05 were used as a level of significance.

## 3. Results

**Figure 2:**
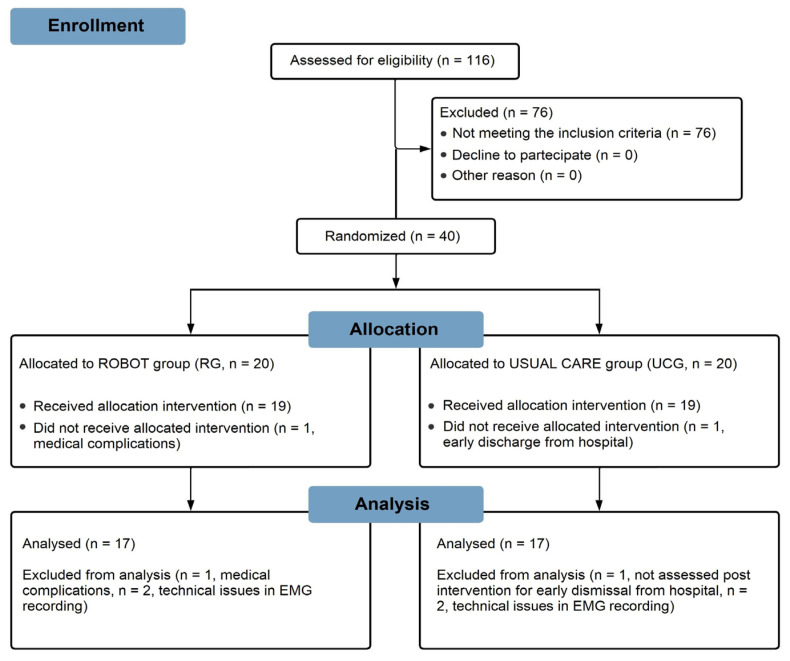
Flow chart of the study.

Thirty-four persons post-stroke, both in chronic and sub-acute stages, completed the object-placing task without technical issues in the sEMG recording and were, therefore, considered for the present analysis: 17 of them were assigned to the RG and 17 to the UCG. A flow chart of the protocol is shown in Figure 2.

### 3.1. Baseline Assessment

The demographic and clinical features of persons post-stroke did not differ significantly between RG and UCG (Table 1).

The median FM–UE score (1st–3rd quartile) at *T*0 was 31.0 (13.0–49.3), indicating a level of disease severity ranging from mild to severe. The healthy subjects were six females and four males with a median age (1st–3rd quartile) of 66.0 (51.0–68.0) years. Both gender and age were not significantly different from those of persons post-stroke.

### 3.2. Comparison of the Instrumental Indices between Healthy Subjects and Persons Post-Stroke at T0

**Figure 3:**
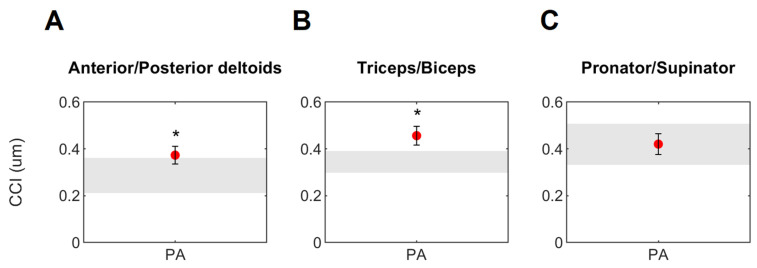
*CCI* of the normative reference (NR, gray band) and the paretic arm (PA, red circles) of persons post-stroke at *T*0 during the object placing task. Circles and whiskers represent, respectively, the mean and 95% confidence interval of *CCI*. * indicates significant differences between NR and PA (*p* ≤ 0.05, unpaired *t*-test).

For the muscle co-contraction during the object-placing task performed at *T*0, the unpaired *t*-test revealed statistically significant differences in *CCI* values between PA and the normative reference (NR) in the anterior/posterior deltoids (*p* = 0.03, Cohen’s d = 0.83, Figure 3A) and triceps/biceps muscle pairs (*p* = 0.01, Cohen’s d = 1.04, Figure 3B). No difference emerged between PA and NR in the pronator/supinator muscle pair (Figure 3C). For the *CCI* nominal values of the two groups, see Table S1 in the Supplementary Materials.

Consistent with the *CCI* findings, the kinematic analysis showed slower movement speed and reduced smoothness in PA compared to NR (see Table S1 in the Supplementary Materials for nominal, *p*-values, and Cohen’s d).

### 3.3. Treatment Effects

No significant clinical effects of treatment were found between UCG and RG from the non-parametric ANCOVA analysis of the FM-UE change score (*p* = 0.15, median (1st–3rd quartile): UCG 4.0 (−0.5–11.0), RG 6.0 (3.0–10.0)) using the FM-UE score at *T*0 as a covariate.

**Figure 4:**
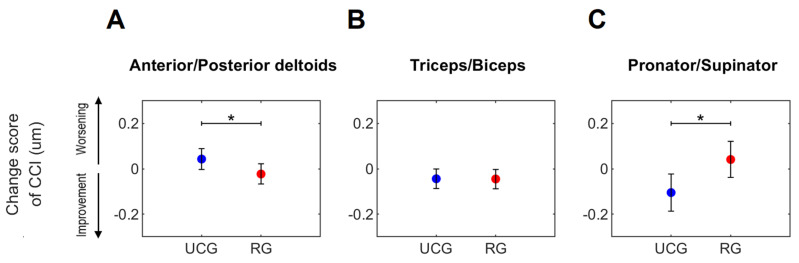
Change score of *CCI* of the paretic arm of persons post-stroke after usual care intervention (UCG, blue circles) and robot therapy (RG, red circles) during the object-placing task. Circles and whiskers represent, respectively, the mean and 95% confidence interval adjusted for *CCI* at *T*0 using the ANCOVA procedure. * indicates significant differences between UCG and RG (*p* ≤ 0.05, ANCOVA analysis).

The results showed treatment-specific effects on *CCI* in the shoulder (Figure 4A) and forearm regions (Figure 4C). In detail, in the RG, 52.9% of the participants showed a reduction in the *CCI* in the anterior/posterior deltoid muscle pair, while, conversely, most of the participants (70.6%) in the UCG showed an increase. Regarding the change score (Figure 4A), a statistically significant difference was found between groups (*p* = 0.05, Cohen’s d = 0.70), with the RG showing a greater reduction in *CCI*, indicating a reduction in deviation from normative values (i.e., positive effect) (see also Table S2 in the Supplementary Materials). Conversely, there was a reduction in the *CCI* in the pronator/supinator muscle pair in the UCG (76.5%) and an increase in the RG (64.7%). Regarding the change score (Figure 4C), a statistically significant difference was found between the treatment groups (*p* = 0.02, Cohen’s d = 0.85), with the UCG showing a greater reduction in *CCI*, indicating a reduction in deviation from normative values (i.e., positive effect) (see Table S2 in the Supplementary Materials).

No significant treatment effect was found in the *CCI* in the elbow region (Figure 4B). In fact, the *CCI* of the triceps/biceps muscle pair showed a reduction in normative values after training (see Table S2 in the Supplementary Materials) and a negative CS in both groups (Figure 4B).

Both groups, regardless of the type of treatment (UCG vs. RG, speed *p* = 0.54), showed an improvement greater than 10% in speed after rehabilitative interventions (see Table S2 in the Supplementary Materials). For the smoothness parameter, only the RG group showed a reduction in smoothness equal to 10% after training.

### 3.4. Correlation Analysis

Table 2 shows the correlation analysis between the change score of the *CCI* and the CS of the FM-UE clinical scale following rehabilitation interventions. From this analysis, no significant correlation was found between the change scores of muscle co-contraction and those of the FM-UE score.

## 4. Discussion

The aim of this study was twofold as follows: (i) to examine the ability of sEMG-based *CCI* to discriminate motor performance between persons post-stroke and healthy subjects, and (ii) to detect changes induced by motor rehabilitation during an untrained 3D functional task. We compared the sEMG data from a cohort of persons post-stroke who underwent two different rehabilitation approaches.

The main finding of our study was that persons post-stroke showed a greater co-contraction of the proximal antagonistic muscle pairs (i.e., higher values of *CCI*) of the paretic arm, while the distal muscle pair co-activation was not altered. Furthermore, we found that the robotic training induced a greater improvement (i.e., negative *CCI* CS) in the motor control of the shoulder joint compared to the control intervention.

### 4.1. Comparison of the Instrumental Indices between Healthy Subjects and Persons Post-Stroke at T0

The proximal *CCI* was able to detect the altered pattern of muscle activation in the paretic side of persons post-stroke highlighting a deviation from the normative reference (Figure 3A,B). These results are consistent with the widespread scientific evidence that persons post-stroke exhibit higher atypical co-contraction levels between paretic muscle pairs when compared to healthy subjects [3,4,5,6,7].

Although previous studies have reported an altered pattern of muscle activation in the distal paretic region of persons post-stroke with respect to the normative reference [3,4,5], no excessive muscle co-contraction in the forearm region emerged in our study. Specifically, Hammond et al. [3] found an alteration in the muscle co-contraction in the flexor carpi radialis/extensor carpi radialis longus during voluntary isometric contraction, Sheng et al. [4] in the extensor/flexor digitorum during a horizontal task, and Kamper et al. [5] in hand muscles under voluntary isometric, isokinetic, and free-range conditions. All these tasks implied a distal muscle activation, while the task presented here was more demanding for the proximal regions, so the pronator/supinator muscle pair was probably less activated with only the stability role of the forearm segment. As a result, in our analysis, the abnormal muscular coordination pattern emerged primarily in the triceps/biceps and anterior/posterior deltoid muscle pairs, in accordance with our previous findings [1,2].

The identification of a biological signals-based index (e.g., *CCI*) that reflects the pathological pattern is crucial for investigating the degree of deviation from the physiological pattern. Overall, the persons post-stroke have difficulty deactivating the muscles of the paretic arm (i.e., higher *CCI* values) [4]. This motor control alteration affects motor performance, causing inefficiency of movements, as indicated by the reduced speed and smoothness. Therefore, the level of muscle co-contraction compared to normative references can be a useful measure for assessing motor performance and residual motor control in persons post-stroke.

### 4.2. Treatment Effects

Recently, Sheng et al. [4] found that the level of muscle co-contraction is associated with the impairment of the corticospinal tracts (CST), as assessed by measuring the motor-evoked potential, suggesting that excessive muscle co-contraction in the upper limb of persons post-stroke has a cortical origin. The abnormality of muscle coordination is linked to the loss of corticospinal projections, and consequently, rehabilitation treatments that enhance the excitability of the CST, especially in subjects with central nervous system injuries (like stroke), could lead to the recovery of a more physiological pattern of muscle activation [4].

In this context, the *CCI* and the analysis presented here could be a suitable tool both for deciding the most appropriate rehabilitation approach and for assessing possible rehabilitation-induced changes. Indeed, global assessments of the motor function and performance improved after training as measured by the FM-UE scale and spatio-temporal kinematic parameters; however, neither evaluation method highlighted any significant treatment effect on upper limb functionality, indicating low responsiveness of both [39]. Conversely, EMG-based *CCI* (Figure 4) was able to detect changes in muscle co-contraction between pre- and post-rehabilitation. Furthermore, these changes were treatment-specific (RG or UCG, see Figure 4), in agreement with a previous study [2]. In detail, the ANCOVA analysis showed that robotic rehabilitation led to a reduction in the *CCI* value of the shoulder and elbow muscles (proximal improvement) and an increase (distal worsening) in the co-contraction of the forearm muscles, whereas conventional therapy induced a reduction in the *CCI* in the forearm region (distal improvement) and an increase in the shoulder joint (proximal worsening). Consistent with our study, Hu et al. [25] also demonstrated a reduction in the abnormal muscle co-contraction in the proximal regions (i.e., shoulder and elbow joints) following robot-assisted elbow training.

The negative effects of rehabilitation treatments should be considered when optimizing future rehabilitation programs. The distal excessive co-contraction developed after the robot treatment could be linked to the design of the robot itself, which, being planar, is mainly focused on shoulder/elbow coordination. As planar robots constitute a standard paradigm for upper limb rehabilitation, the design of these devices should be modified to achieve a greater involvement of the distal segment, or additional exercises of the distal region should be administered in case of using planar robots. The excessive shoulder co-contraction following conventional therapy could be related to a less precise and repeatable, and therefore less effective, therapeutic effect [40] compared to the robotic therapy, on the well-known problems of shoulder impairments after stroke both in terms of glenohumeral joint injury [41] and overuse in the adopted compensatory strategy [42].

The results of this study pointed out that *CCI* can also be a useful biomarker for tailored rehabilitation as it provides suggestions on how to improve and personalize treatments. For example, robot therapy with the Braccio di Ferro manipulandum might be ameliorated by combining it with rehabilitation exercises (conventional or even robot-assisted) specifically targeted at the wrist/hand muscles.

The present results on muscle overactivity are in line with our previous study [2], in which we found comparable rehabilitation-induced changes in muscle synergies activation patterns [43] using a more complex evaluation (i.e., the motor control was decomposed into a limited number of primitive patterns using clustering techniques), whose extraction and interpretation require high technical expertise and is, therefore, difficult to implement in clinical practice. Conversely, the use of an easily computable and intuitive index such as *CCI* seems to be a more feasible approach for direct clinical application.

As highlighted by our findings, the *CCI* value detects the changes in the upper extremity motor function, indicating that it could be used to support clinical assessment and monitor motor improvement during stroke recovery [4]. In fact, the sEMG-based assessment of muscle co-contraction has recently been included for the benchmarking of upper limb functions in neurological disorders [44]. Indeed, the authors of this study [44], which focused on standardizing the evaluation of intra-limb coordination and identifying a quantitative and high-resolution metric, selected the *CCI*s as performance indicators (i.e., “outcome measure that allows the quantitative assessment of a motor ability” [44]).

Traditionally, the assessment of arm motor function and the effectiveness of rehabilitation treatments rely on standard clinical scales [44]. Our analysis of the correlation between the rehabilitation-induced changes in *CCI* value and FM-UE score (Table 2) showed no association between these two measures, consistent with the findings of other studies [4,44]. On the contrary, Chae et al. [45] and Chalard et al. [46] found a negative correlation between the level of co-contraction and upper limb motor functionality (i.e., indicated by the score of FM-UE). This incongruence was probably related to the different experimental conditions from which the sEMG signal was recorded; the above-cited studies examined the muscle co-contraction in an isometric condition of single-joint movements, whereas we assessed muscle concentric activation during a goal-directed movement, which implied a 3D dynamic coupling and coordination of the multi-joint system. FM-UE measures gross and isolated single-joint movements; therefore, the presence of correlation between *CCI* and FM-UE is more probable in isometric conditions than in a task that requires coordination, such as the one performed here.

Moreover, clinical scales have some metric characteristics (e.g., measurement of ordinality, and possible floor and ceiling effects) that might limit their reliability and responsiveness, particularly when compared with an objective, instrument-based, and continuous measure, like *CCI*.

In persons post-stroke, the features expressed in most functional movements, such as the task presented here, are the direct consequence of the lesion to the CST and/or somatosensory cortex: (i) loss of individual articular joint movements, (ii) general slowness with temporal irregularities and jerkiness, (iii) alterations in the somato-motor reactive control with time delays, and (iv) disrupted inter-joint muscle coordination in the upper limb.

In this framework, our results proved that EMG-based *CCI* provides additional information for a deeper understanding of muscle coordination impairments. Since the recovery at the biological level, particularly with respect to the CST, is an important vector for the return of upper limb motor control, such an index could be a promising tool for selecting proper rehabilitation treatment [22,44], increasing functional recovery [17], and improving the reliability and reproducibility of studies that evaluate the effectiveness of clinical interventions [18].

### 4.3. Study Limitations

This study does have some limitations. First, sEMG assesses the final peripheral pathway, although we expect sEMG signals to also provide information about motor coordination. A second limitation is the lack of follow-up assessments that did not allow the analysis of the retention of training effects.

Finally, the inclusion of distal robotic components should also be addressed to possibly enhance the effect on the co-contraction of wrist muscles and promote a better transfer to ADLs.

Aware of the above limitations, future studies with a larger sample size should be performed to corroborate the present findings, including EEG/fMRI evaluations, and to assess long-term training effects.

## 5. Conclusions

Quantifying upper limb motor functionality is essential to comprehend and monitor neuromotor recovery [44,47]. Our study indicates that sEMG-based *CCI* may be a promising quantitative index to assess motor deficit levels and rehabilitation-induced changes in motor skills of persons post-stroke. Its capacity to describe changes in motor coordination is important for a more complete motor assessment of arm functionality post-stroke and might be of benefit for tailored rehabilitation leading to improved arm use in daily life.

## Figures and Tables

**Table 1:** Demographic and clinical characteristics of persons post-stroke allocated to robot and usual care groups.

Variable		UCG (N = 17) Median (1st–3rd)	RG (N = 17) Median (1st–3rd)	*p*-Value
Age (years)		59.0 (46.0–70.0)	67.0 (58.0–72.0)	0.20
Time since stroke (months)		5.8 (1.9–91.4)	7.8 (1.4–13.9)	0.55
FM-UE		21.0 (12.0–46.5)	33.0 (16.0–50.5)	0.22
		**Number**	**Number**	
Sex				0.73
	Female	8	9	
	Male	9	8	
Stroke type				1.00
	Ischemic	11	11	
	Hemorrhagic	6	6	
Paretic side				0.49
	Right	6	8	
	Left	11	9	
Chronicity (>3 months)				1.00
	Chronic	10	10	
	Sub-acute	7	7	

UCG: Usual care group. RG: Robot group. FM-UE: Fugl-Meyer Motor Assessment for the Upper Extremities. *p*-values indicate the results of the Mann–Whitney U Test for age, time since stroke, and FM-UE baseline score and of the chi-square tests for all the other variables.

**Table 2:** Spearman’s correlation analysis between the change scores of *CCI* and FM-UE clinical scale.

		FM-UE CS	
		Correlation Coefficient	*p*-Value
**CCI CS**	Anterior/Posterior deltoids	0.04	0.83
	Triceps/Biceps	0.03	0.85
	Pronator/Supinator	–0.09	0.63

CS: change scores. *CCI*: Frost’s co-contraction index. FM-UE: Fugl-Meyer Motor Assessment for the Upper Extremities. *p*-values indicate the results of Spearman’s correlation.

## Data Availability

The dataset used and/or analyzed during the current study are available from the corresponding author upon reasonable request.

## Supplementary Materials

The following supporting information can be downloaded at: https://www.mdpi.com/article/10.3390/s23177320/s1, Table S1. Mean and 95% confidence interval of *CCI* and of kinematic parameters of healthy subjects and paretic arm of persons post-stroke, before rehabilitation treatment (*T*0), during the object placing task; Table S2. Mean and 95% confidence interval of *CCI* and of kinematic parameters of healthy subjects and paretic arm of persons post-stroke, before (*T*0) and after (*T*1) rehabilitation treatment, during the object placing task.
