# Supplementary material for: Surface-Electromyography-Based Co-Contraction Index for Monitoring Upper Limb Improvements in Post-Stroke Rehabilitation: A Pilot Randomized Controlled Trial Secondary Analysis
Source: Sensors (Basel). 2023 Aug 22;23(17):7320. doi: 10.3390/s23177320 (PMC10490112; doi:10.3390/s23177320)
Supplement: Supplementary file 1 [file sensors-23-07320-s001.zip › sensors-2529155-supplementary.pdf]

Supplementary Materials

Table S1. Mean and 95% confidence interval of CCI and of kinematic parameters of healthy subjects and paretic arm of persons post-stroke, before rehabilitation treatment (T0), during the object placing task.

		**NR (N=10) Mean (95% CI)**	**PA (N=34) Mean (95% CI)**	**P-value**	**Cohen's d**
**CCI**	Anterior/Posterior deltoids	0.29 (0.21–0.36)	0.38 (0.34–0.41)	0.03*	0.83
	Triceps/Biceps	0.34 (0.30–0.39)	0.46 (0.41–0.50)	0.01*	1.04
	Pronator/Supinator	0.42 (0.33–0.51)	0.42 (0.38–0.47)	0.96	0.02
**Kinematic parameters**	Speed (m/s)	0.199 (0.155–0.243)	0.109 (0.082–0.136)	<0.01*	1.18
	Smoothness (peaks/mm)	0.08 (0.06–0.10)	0.19 (0.16–0.22)	<0.01*	1.44

* NR: healthy subject. PA: paretic arm of persons post-stroke. CI: confidence interval. The CCI ranges from 0 to 1 (dimensionless). The speed is reported in m/s, while the smoothness in peaks/mm. * indicates significant difference between PA and NR (P≤0.05, unpaired t-test).

Table S2. Mean and 95% confidence interval of CCI and of kinematic parameters of healthy subjects and paretic arm of persons post-stroke, before (T0) and after (T1) rehabilitation treatment, during the object placing task.

		**NR (N=10) Mean (95% CI)**	**UCG (N=17) Mean (95% CI)**		**RG (N=17) Mean (95% CI)**	
			**T0**	**T1**	**T0**	**T1**
**CCI**	Anterior/Posterior deltoids	0.29 (0.21–0.36)	0.39 (0.33-0.45)	0.42 (0.37-0.48)	0.36 (0.31-0.41)	0.34 (0.29-0.40)
	Triceps/Biceps	0.34 (0.30–0.39)	0.45 (0.39-0.51)	0.41 (0.36-0.46)	0.46 (0.40-0.52)	0.41 (0.36-0.46)
	Pronator/Supinator	0.42 (0.33–0.51)	0.45 (0.38-0.53)	0.32 (0.24-0.40)	0.39 (0.33-0.45)	0.46 (0.37-0.54)
**Kinematic parameters**	Speed (m/s)	0.199 (0.155–0.243)	0.108 (0.062–0.154)	0.140 (0.086–0.193)	0.110 (0.077–0.144)	0.125 (0.083–0.166)
	Smoothness (peaks/mm)	0.08 (0.06–0.10)	0.20 (0.15–0.25)	0.21 (0.11–0.32)	0.18 (0.15–0.21)	0.16 (0.12–0.21)

* NR: healthy subject. UCG: usual care group. RG: robot group. T0: pre-training. T1: post-training. CI: confidence interval. The CCI ranges from 0 to 1 (dimensionless). The speed is reported in m/s, while the smoothness in peaks/mm.
